# Effects of Substrate Roughness on Microstructure and Fatigue Behavior of Plasma Electrolytic Oxidation-Coated Ti-6Al-4V Alloy

**DOI:** 10.3390/ma15124256

**Published:** 2022-06-15

**Authors:** Fangquan Xi, Yong Huang, Yahui Zhao, Yang Liu, Weibing Dai, Yanzhong Tian

**Affiliations:** 1 School of Mechanical Engineering & Automation, Northeastern University, Shenyang 110819, China; 2110097@stu.neu.edu.cn (F.X.); 2070141@stu.neu.edu.cn (Y.H.); 1970248@stu.neu.edu.cn (Y.Z.); 2 Key Laboratory of Vibration and Control of Aero-Propulsion System Ministry of Education, Northeastern University, Shenyang 110819, China; 3 School of Mechanical Engineering, Liaoning Technical University, Fuxin 123000, China; yifanwb@163.com; 4 Key Laboratory for Anisotropy and Texture of Materials (Ministry of Education), School of Materials Science and Engineering, Northeastern University, Shenyang 110819, China; tianyanzhong@mail.neu.edu.cn

**Keywords:** roughness, microstructure, fatigue behavior, fatigue behavior, stress concentration

## Abstract

Ceramic coatings were prepared by plasma electrolytic oxidation (PEO) on four different surface roughness’ of Ti-6Al-4V alloys. The effects of substrate roughness on the microstructure and fatigue behavior were investigated. Microstructural characterization was carried out by scanning electron microscopy (SEM) and a laser scanning confocal microscope. In addition, an X-ray diffractometer (XRD) and a U-X360 stress meter were used to analyze the phase composition and residual stress properties of the coatings. The microstructure of coatings revealed the growth mechanism of the coatings. The larger and deeper grooves of the substrate promoted the nucleation and growth of the PEO coating, but the defects (cracks and pores) of the oxide layer became more serious. The fatigue test indicated a significant influence of substrate roughness on the fatigue life under low cyclic stress. The fatigue damage of PEO coatings decreases as the surface roughness of substrates decreases because of the synergistic effect of the coating surface defects and coating/substrate interface roughness. Substrate roughness influences the quality and fatigue performance of the oxide layer.

## 1. Introduction

Ceramic coatings have extensive applications in cutting tools due to the higher hardness, wear resistance, and thermal stability [1]. For example, Al_2_O_3_-coated tools can effectively improve machining efficiency and machining accuracy [2,3]. The methods of commonly making ceramic coatings are chemical vapor deposition (CVD) and physical vapor deposition (PVD) [4]. Recently, plasma electrolytic oxidation (PEO) is a new process for in situ ceramic coatings on the surface of alloys. While the oxide layers improve the wear and corrosion resistance, the oxidation treatment can significantly reduce the fatigue performance of the alloy [5,6]. The PEO coating has a poor fatigue strength, which restricts its application [7,8,9]. Therefore, it is significant to study the fatigue fracture mechanism of coated alloys to promote the wide application of the PEO coating in the chemical and mechanical manufacturing industries.

Some studies showed that substrate roughness would be known to affect the hardness, corrosion, and wear resistance of PEO coatings [9,10,11]. Yoo et al. [9] suggested that the corrosion resistance of coatings degraded as the surface roughness increased. Wang et al. [12] concluded that the micro-groove pretreatment could dramatically improve the hardness and wear resistance of coated samples. Ni et al. [7] reported that the ultrasonic surface rolling process (USRP) pretreatment improved the coating compactness because the smoother substrate surface was beneficial for improving the distribution uniformity of the pores of the coatings. While surface roughness was a crucial factor affecting the corrosion and wear resistance of the oxide layer, the effect of substrate roughness on the mechanical properties and the fatigue life of coated samples is rarely researched. Therefore, the following questions need to be resolved:

(a) Although it has been found that reducing the substrate roughness is beneficial to improve the corrosion resistance of the oxide layer [12,13], whether or not such the treatment can improve the fatigue performance of coatings needs to be further researched.
(b) How does substrate roughness affect coating microstructure?
(c) Although the mechanism for the reduction of the fatigue properties of oxide layers has been reported, whether or not this mechanism is still valid when the substrate roughness changes needs to be verified.

Towards meeting these objectives, ceramic coatings were prepared by plasma electrolytic oxidation (PEO) on four different surface roughness of Ti-6Al-4V alloys. The coatings were analyzed for the surface and the cross-sectional morphologies, phase composition, and residual stress properties. The fatigue performance of all substrate and coated samples was evaluated under two different pre-calculated stress levels. The effects of substrate roughness on fatigue strength and the morphologies of oxide layers were investigated.

## 2. Experimental

### 2.1. Preparation of Substrate Samples

**Figure 1:**
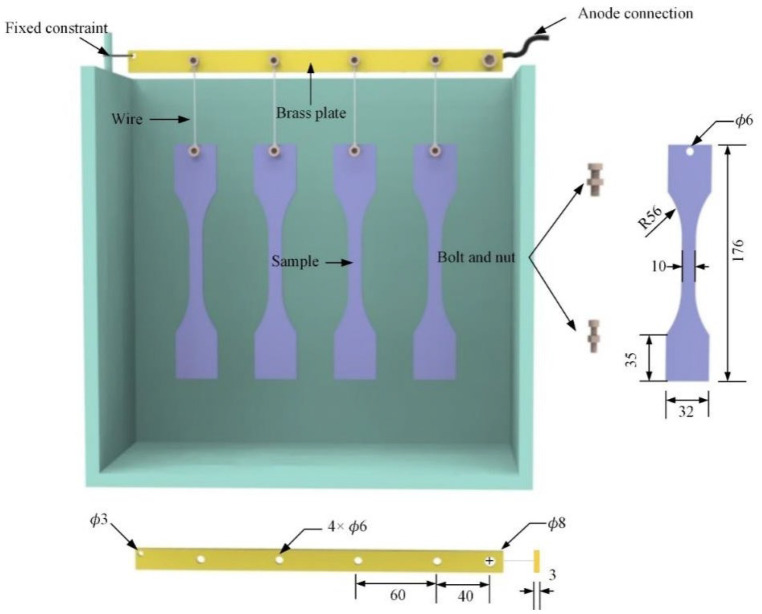
The schematic diagram of samples and fixture assembly.

**Figure 2:**
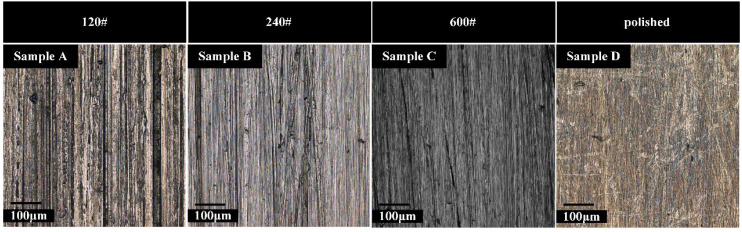
Surface morphologies of substrate samples: Substrate roughness of (**A**) *R_a_*3.2, (**B**) *R_a_*1.6, (**C**) *R_a_*0.8, and (**D**) *R_a_*0.2.

The size of substrate samples is illustrated in Figure 1. Before PEO treatment, substrate samples were polished by grit sizes of 120#, 240#, 600#, and 1000# Sic papers and followed by W3.5 diamond suspension to ensure samples to a polished surface. The processed Ti-6Al-4V alloy samples were divided into four groups that were marked as the samples A, B, C, and D, respectively (Figure 2). Substrate samples were ultrasonically cleaned in ethanol and dried with air oven.

### 2.2. Preparation of Coating on Ti-6Al-4V by PEO Treatment

The PEO treatment was prepared on the substrates through a bipolar pulsed 65 kW plasma electrolytic oxidation device (Institute of Special Ceramics, Harbin Institute of Technology, Harbin, China). PEO coatings were deposited under a fixed DC voltage of 400 V, duty cycle and oxidation time of 10% and 10 min, respectively. The electrolyte consisted of 10 g/L Na_2_SiO_3_ and 20 g/L (NaPO_3_)_6_ in 1 L of distilled water with the stainless-steel plate cathode, and samples were used as the anode. After PEO treatment, the coated samples were cleaned with deionized and dried in an air oven [14,15].

### 2.3. Microstructure of Coatings

The three-dimensional (3D) morphologies of samples were observed via laser scanning confocal microscope (LEXT OLS 4100, Analysis and Testing Center, Northeastern University, Shenyang, China). According to the ISO 4287 standard, surface roughness was measured by LEXT OLS 4100 instrument analysis software. The surface and cross-section morphologies of PEO-coated samples were observed by SEM, and the coated thickness was measured by SEM (ten positions were selected randomly for each coated sample along the axis gauge length (100 µm), and the average of five data were used as the coated thickness). The mean diameter of pores and porosity of coatings were calculated by the Image J analysis software. To ensure the accuracy of experimental data, 3–5 areas of SEM images were measured with the same unit area. The elemental distributions of coated samples were determined by the energy dispersive X-ray spectrometer (EDS).

### 2.4. Phase Composition of Coatings

The phase compositions of PEO coatings were determined by XRD-7000s type X-ray diffraction (Cu-Kα). The equipment operated under an accelerating voltage of 40 kV and a current of 40 mA. The coated samples were scanned by grazing incidence with the grazing angle, scanning angle, and scanning speed of 0.7° (in *θ*), 10–90° (in 2*θ*), and 2°/min, respectively.

### 2.5. Residual Stresses of Coatings

The residual stresses of substrate and PEO-coated samples were measured by the U-X360-stress analyzer (Cu-K) based on the X-ray diffraction Debye ring analysis. Because the obvious peak value usually appeared when the diffraction angle was small in the thin film and coating [16,17], the incidence angle was set to 23.8°. The diffraction angle of 117.79° and 213 surface conditions of diffraction crystal were determined. Young’s modulus (*E*) and Poisson’s ratio (*ν*) of TiO_2_ and Ti were set at 113 GPa, 0.32 and 180 GPa, 0.26, respectively [6,7].

### 2.6. Fatigue Properties of Coatings

The fatigue strength of substrate and PEO-coated samples was performed for axial loading using the EHF-EV200K2-040-1A (School of Mechanical Engineering & Automation, Northeastern University, Shenyang, China). The loading frequency and a stress ratio were set to 10 Hz and *R* = 0.1, respectively. The effect of PEO treatment on fatigue behavior cannot be predicted before the fatigue test. To study the effect of the PEO treatment on the high cyclic fatigue life and low cyclic fatigue life, the fatigue life of the PEO coated samples should be changed significantly. Therefore, the stress levels of fatigue tests are based on the fatigue life of *R_a_* = 0.2 substrate samples. The stress corresponding to the fatigue life of *R_a_*0.2 substrate reaching more than 10^5^ is regarded as the high cycle fatigue condition and that of fatigue life under 10^5^ as the low one (850 MPa). Three fatigue samples were evaluated under the same stress level considering the accuracy of the data.

## 3. Results and Discussion

### 3.1. Microstructure of PEO Coatings

**Figure 3:**
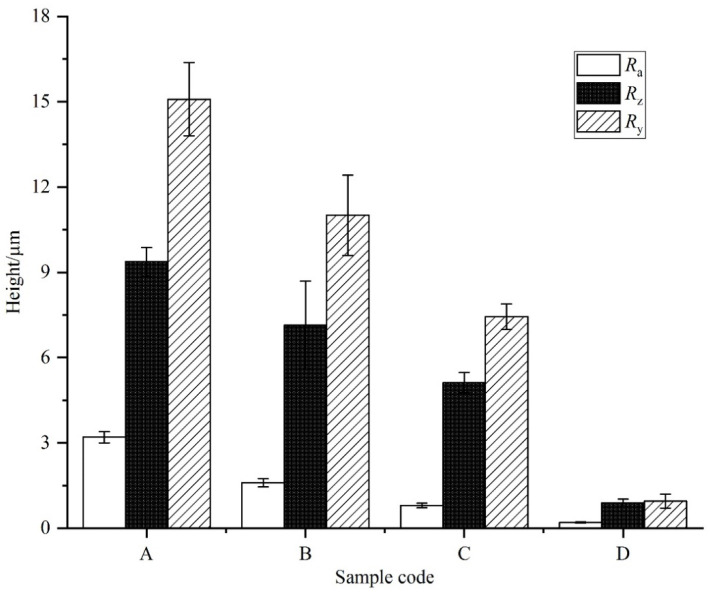
The two-dimensional roughness parameters of substrate samples.

According to the ISO 4287 standard, two-dimensional roughness parameters of *R_a_*_,_ *R_y_*, and *R_z_* were measured. Specifically, these parameters are closely related to the stress concentration factor *K_t_*, and the influence of *K_t_* on fatigue performance is well known [18]. Therefore, it is reasonable to use them to quantify the surface profile of the substrate. As can be shown in Figure 3, the sample D has the lowest roughness value.

**Figure 4:**
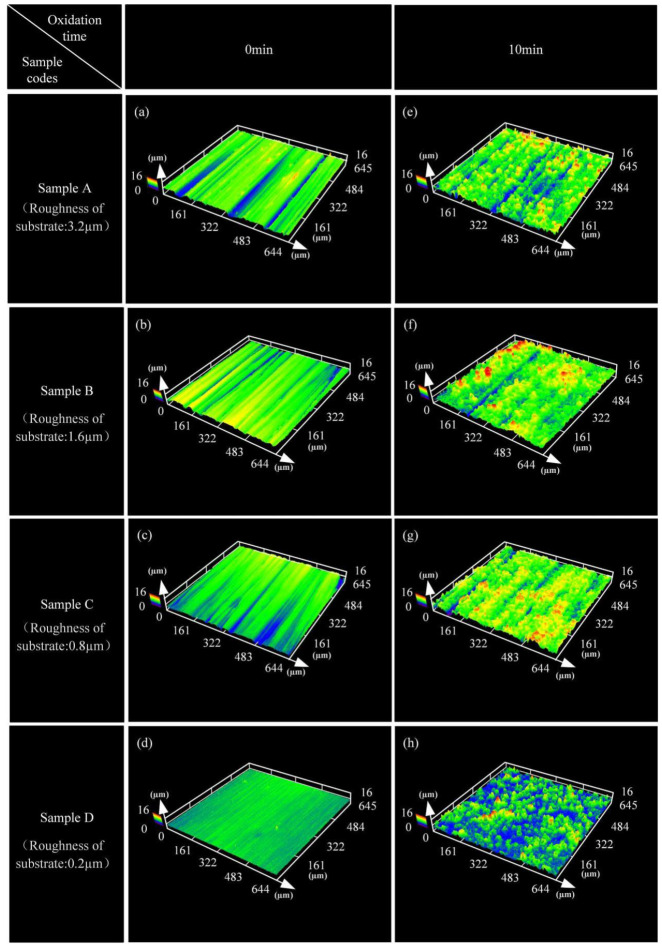
3D morphologies of the substrate (**a**–**d**) and PEO-coated samples (**e**–**h**).

Figure 4 shows the 3D surface morphologies of PEO coatings. The different colors of the 3D surface morphology characterize different morphological heights. The contour of the grooves is visible on the substrate surface, which are caused by grinding before PEO treatment. After PEO treatment, the samples became smooth, because the coating filled the grooves on the surface of the substrate. However, there are still some shallow grooves on the surface of coated samples A and B, and they are not evenly distributed homogeneously. This fact implied that the growth rate of the coatings was expected to be different in the samples with different surface roughness.

**Figure 5:**
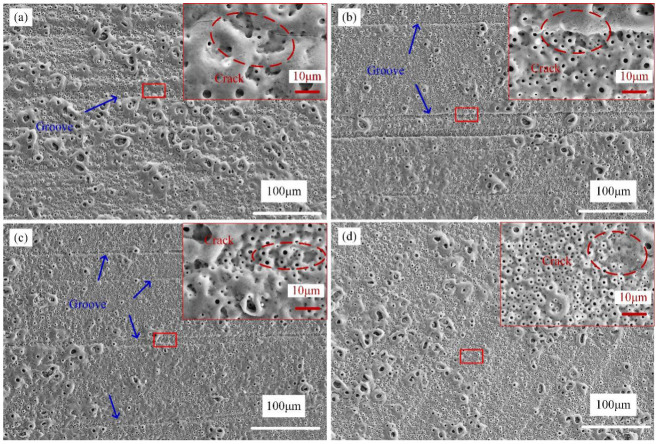
Surface morphology of PEO-coated samples: Substrate roughness of (**a**) 3.2; (**b**) 1.6; (**c**) 0.8; and (**d**) 0.2 µm.

Figure 5 shows the surface morphologies of PEO-coated samples. Although a large number of mic-pores and mic-cracks occurred in four samples, the distribution uniformity of the pores is different. Small pores (SP) and a small number of large pores (LP) are uniformly distributed on the surface of samples C and D. However, a large number of LP are around the valleys for samples A and B. The study proved that LP were characteristic of the rapid growth of coatings [19,20,21].

The growth mechanism of PEO coatings was divided into three steps, which were the breakdown step, the coatings formation step, and the coating melting step [20,22]. The SP formed in the second step. Due to the electrolysis of H_2_O, gas bubbles discharged the molten oxide that were deposited onto the surface. It led to the formation of the coating, while the channels formed holes on the surface. The LP formed in the third step. The main reason for the formation of LP was that the oxide between a part of channels at a close distance was melted and deposited again. Therefore, the phenomenon of LP and SP was a sign of non-uniformity coating growth. Because the increase in substrate roughness resulted from a steeper peak–valley structure, which was of benefit to plasma aggregation, therefore, the surface of sample A and B entered the third step earlier than sample C and D. As can be seen in Figure 5a,d, LP had a clustered distribution on sample A and an even distribution on sample D. The clustered distribution of LP was the sign of accumulation of micro-discharges. This phenomenon suggested that the morphology of each area on the rougher surface was quite different, which was more likely to cause the accumulation of micro-discharges. The porosity and mean pore diameter of the coatings were reflected in Table 1. The porosity and mean pore diameter of the coated sample A are all higher than that of others. The results indicate that the rough surface of the substrate increases the non-uniformity of micro-discharges.

**Figure 6:**
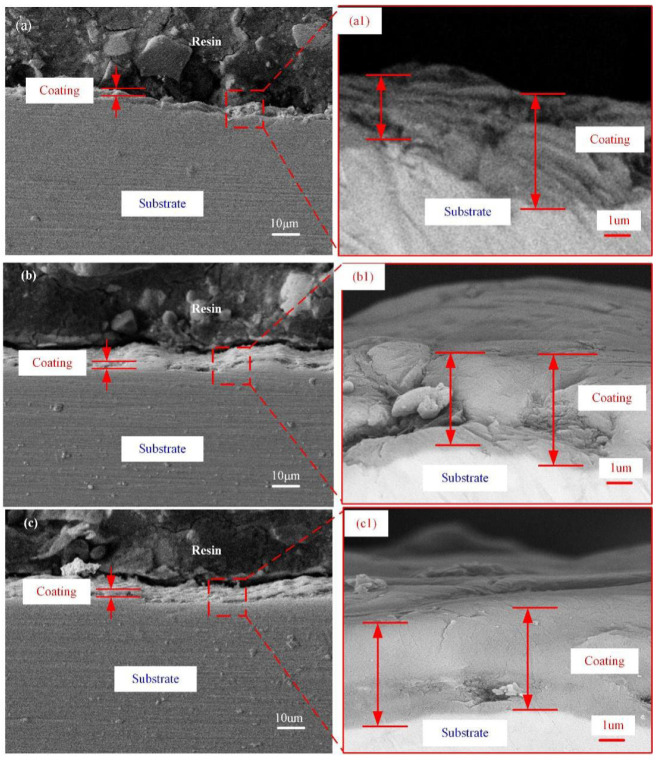

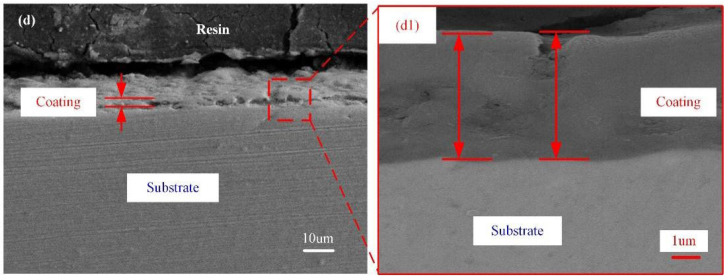
Cross-sectional morphology of PEO-coated samples: Substrate roughness of (**a**,**a1**) 3.2; (**b**,**b1**) 1.6; (**c**,**c1**) 0.8; and (**d**,**d1**) 0.2 µm.

The coated thickness was measured by taking many locations of the cross-section. As presented in Figure 6(d1), compared to other samples, the coated sample D shows the more uniform thickness of the oxide layer. N. Nashrah et al. [13] reported that if micro-discharges take place evenly throughout the surface of the substrate, the oxide layer of the sample grows uniformly. As surface roughness affected the size distribution of micro-discharges, the oxide layers of the different growth rates were in different surface roughness. To express the uniformity of the coating thickness, the thickness non-uniformity factor *T_d_* was introduced.

(1) $$T_{d}=\left(H_{2}-H_{1}\right)/H_{2}$$

*H*_2_ and *H*_1_ are the maximum and minimum values of coating thickness, respectively. The coating thickness and the thickness non-uniformity factor *T_d_* are listed in Table 1. The thickness uniformity of four coated samples was significantly different. As the substrate roughness increases, the thickness uniformity of PEO coatings becomes worse. This finding suggested that the lower the substrate roughness is, the more homogeneous the micro-discharges distribution is. Thus, the thickness of the coated sample D was more uniform than that of others.

### 3.2. Residual Stress on the Surface of the Substrate and PEO Coating

Some studies have shown that the decrease in fatigue performance of PEO coatings is greatly related to the residual stress, produced in the growth of the PEO coating [7,14]. To be specific, there are two main reasons for the generation of the residual stress. One is the difference between the thermal expansion coefficients of the coating and the substrate. The other is the temperature gradient in the partial discharge area of the coating [17].

**Figure 7:**
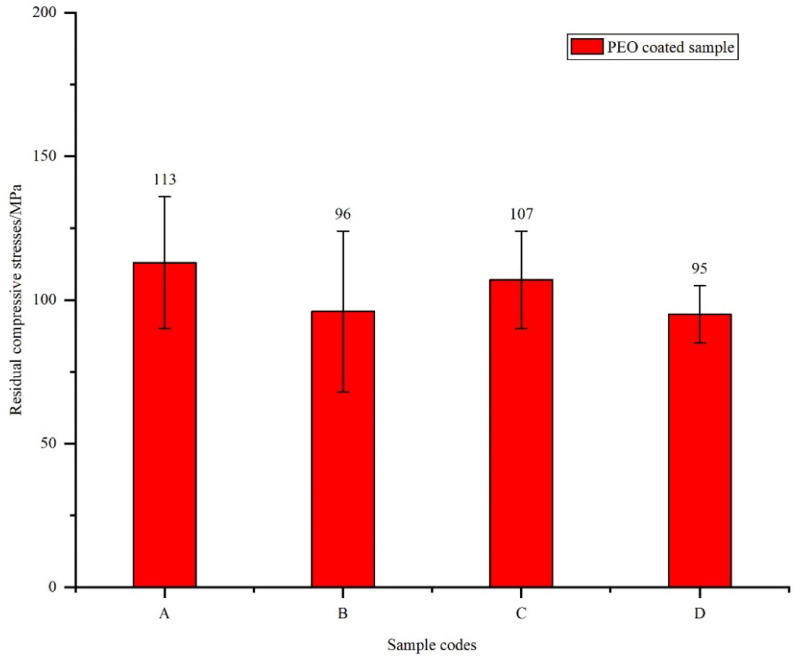
Residual compressive stress of the PEO-coated samples: Substrate roughness of (A) 3.2, (B) 1.6, (C) 0.8, and (D) 0.2 µm.

The surface roughness has little effect on the residual stress properties of the PEO coating surface, as shown in Figure 7. Ni et al. [7] found that when machining residual stresses (200 ± 28 MPa) exist on the surface of substrates, tensile stresses of the substrate interface generated by the PEO treatment can be eliminated. In the present study, the large residual compressive stress (−207 ± 4 MPa) exists on the surface of the original substrate. Therefore, the machined-induced residual compressive stress can effectively suppress the effect of the residual tensile stress, generated on the surface of the substrate by the PEO treatment, on the fatigue performance.

### 3.3. Phase Compositions of PEO Coating Samples

**Figure 8:**
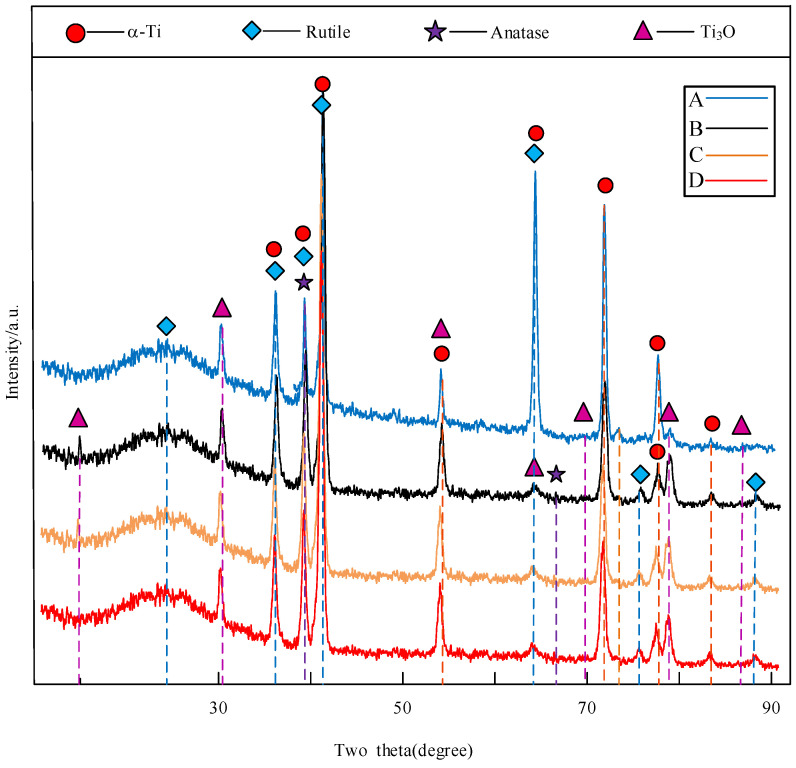
XRD patterns of PEO coatings: Substrate roughness of (A) 3.2, (B) 1.6, (C) 0.8, and (D) 0.2 µm.

Ceramic coatings were mainly composed of rutile, anatase, Ti_3_O, and plenty of small amorphous materials as shown in Figure 8. The rutile, Ti_3_O, and anatase phases, respectively, conform to ICSD 63710, ICSD 23575, and ICSD 31024 [23]. The diffraction peaks of titanium of PEO-coated sample A are more obvious at 2*θ* = 63.5°, compared with other samples. Section 3.1 shows that although the average thickness of PEO-coated samples has little difference, the thickness uniformity of the coated sample A is poor. The thinner thickness and more pores of the coating make X-rays easily penetrate the coating [24,25].

### 3.4. Fatigue Performance of Substrate and PEO Coatings

**Figure 9:**
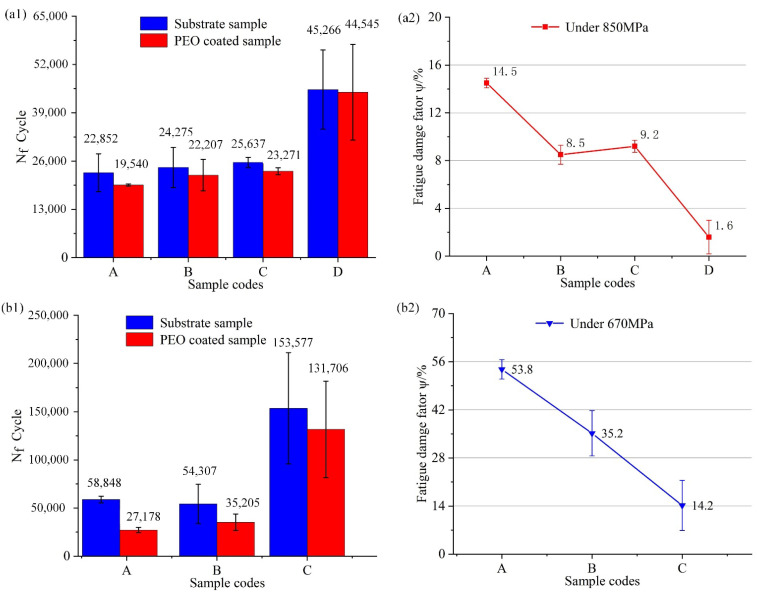
Fatigue strength of the substrate and PEO-coated samples: Substrate roughness of (A) 3.2, (B) 1.6, (C) 0.8, (D) 0.2 µm. (**a1**) Fatigue life of substrate and PEO-coated samples under 850 MPa. (**a2**) Fatigue damage factor *ψ* under 850 MPa. (**b1**) Fatigue life of substrate and PEO samples under 670 MPa. (**b2**) Fatigue damage factor *ψ* under 670 MPa.

The fatigue life for the substrate and the coated samples at *σ*_max_ = 850 MPa, *σ*_max_ = 670 MP as shown in Figure 9. To indicate the damage degree of PEO coatings to substrates, the fatigue variable parameter *ψ* is efined as:

(2) $$\psi =\left(N_{f}-N_{f^{\prime }}\right)/N_{f}$$

where *N_f_* is the average fatigue life of the substrate sample, and *N_f_*_′_ is that of the PEO-coated sample.

The fatigue damage of four coated samples was all less than 15% as shown in Figure 9(a2). The main reason for this phenomenon was that the crack propagation stage was the main stage of the fatigue fracture process under high cyclic stress. To conclude, substrate roughness has a small effect on the fatigue damage rate at high cyclic stress levels. When *σ*_max_ was 670 MPa, the data about sample D was not illustrated in Figure 9(b2) because the substrate and the PEO-coated sample D did not fracture in 10^6^ cycles. Compared with high cyclic stress, low cyclic stress had a more marked effect on fatigue life. Especially the fatigue damage of sample A that was 53.8%, because the fatigue crack initiation occupies most of the fatigue life [25,26]. The coating defects (cracks) of sample D are fewer than that of others because the surface of the smooth substrate reduces the local discharge phenomenon. Therefore, sample D has a longer crack nucleation life than that of others under the cyclic loading.

**Figure 10:**
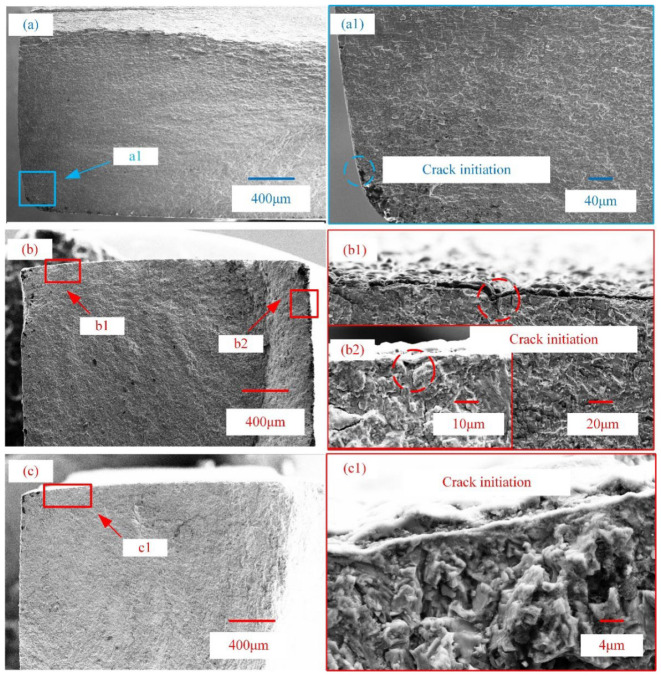
Fatigue morphologies of substrate sample A (**a**,**a1**), PEO-coated samples A (**b**,**b1**,**b2**) and C (**c**,**c1**) under 670 MPa.

Figure 10 shows the fracture morphologies of substrate and PEO-coated samples when the maximum alternating stress is 670 MPa. As shown in Figure 10(b1,b2), the fatigue crack initiations are mainly concentrated on valleys of the coating surface (marked by circles). The fatigue crack of the substrate was initiated by the processing defect (Figure 10(a1)). Compared with PEO-coated sample C, the coated sample A had multiple cracks as shown in Figure 10b. As the fatigue crack is initiated from the surface defects of the PEO coating [7,14], the poor surface quality of coated sample A leads to easy cracking under the cyclic loading. The coating/substrate interface has no obvious delamination, which facilitated the penetration of coating cracks into the substrate as shown in Figure 10(b1). A slight plastic deformation occurred in the substrate near the coating/substrate interface zone, because the crack propagation ran through the interface into the substrate from the coating (Figure 10(b2)). The results indicated that the synergistic effect of coating surface defects and the interface promotes the initiation and growth of fatigue cracks.

**Figure 11:**
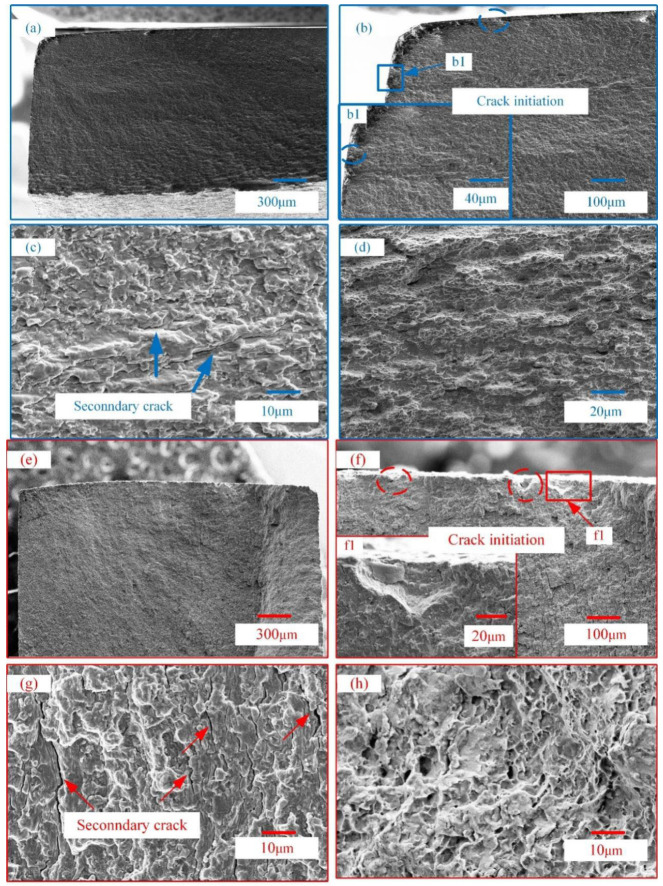
Fatigue morphologies of substrate sample A (**a**,**b**,**b1**,**c**,**d**) and PEO-coated sample A (**e**,**f**,**f1**,**g**,**h**) under 850 MPa.

The fatigue fracture morphologies of the substrate and PEO-coated sample A under 850 MPa are shown in Figure 11a. The fatigue process goes through three stages, the crack initiation stage (Figure 11b,f), the crack propagation stage (Figure 11c,g), and finally the fracture (Figure 11d,h). The crack initiation of the substrate may be caused by the stress concentration. Figure 11f shows that fatigue cracks are mainly concentrated on valleys of the surface of the coating. The valleys produce a large stress concentration under the action of high cyclic stress, which promotes the initiation of fatigue cracks.

The fatigue cracks on the different levels extend from the surface of the coating to the inside of the substrate along the slip surface [27,28], which leads to the crack propagation region of the coated sample having a step morphology, as shown in Figure 11e. The final fracture region of the substrate and coated samples is different. Compared to the substrate, the coated sample has a rough fracture surface due to multiple cracks on the coating surface.

## 4. Conclusions

The effect of substrate roughness on the formation and fatigue performance of PEO coatings is investigated in the present study. The following are the main conclusions:

(1) Substrate roughness influences the formation of PEO coatings. Although the growth rate of the rough sample was higher than that of the smooth sample, the defects of the coating become more serious.
(2) Substrate roughness has a small effect on the phase compositions and properties of residual stresses of oxide layers. Although the initial roughness of substrate samples is different, the final roughness of coated samples is more similar.
(3) The fatigue life of the coated Ti-6Al-4V alloy can be improved by reducing the substrate roughness. The fatigue damage of PEO coatings decreases with the surface roughness of substrates decreasing under low cyclic stress, because of the synergistic effect of coating surface defects and coating/substrate interface roughness.

## Figures and Tables

**Table 1:** Porosity, mean pore diameter, thickness, and thickness non-uniformity factor *T_d_* of PEO-coated samples.

PEO Sample	A	B	C	D
Thickness (µm)	3.96 ± 1.09	3.65 ± 0.80	3.80 ± 0.54	3.60 ± 0.50
Td (%)	44.0%	24.3%	18.4%	7.7%
Porosity (%)	5.97	5.20	4.43	4.96
Mean pore diameter (µm)	0.907	0.850	0.865	0.808
Roughness (µm)	1.32 ± 0.13 µm	1.04 ± 0.10 µm	1.05 ± 0.10 µm	1.18 ± 0.12 µm

## Data Availability

Not applicable.

## References

[1] Zhang J., Zhang G., Fan G. (2022). Effects of tool coating materials and coating thickness on cutting temperature distribution with coated tools. Int. J. Appl. Ceram. Technol..

[2] Brito R.F., de Carvalho S.R., Silva S.M.M.D.L.E., Ferreira J.R. (2009). Thermal analysis in coated cutting tools. Int. Commun. Heat Mass Transf..

[3] Gevorkyan E., Rucki M., Panchenko S., Sofronov D., Chałko L., Mazur T. (2020). Effect of SiC Addition to Al_2_O_3_ Ceramics Used in Cutting Tools. Materials.

[4] Sawka A., Kwatera A., Woźnicki A., Zasadziński J. (2016). Cemented carbide cutting tools life with nanocrystalline Al_2_O_3_ layer deposited by MOCVD. Arch. Civ. Mech. Eng..

[5] Lonyuk B., Apachitei I., Duszczyk J. (2007). The effect of oxide coatings on fatigue properties of 7475-T6 aluminium alloy. Surf. Coatings Technol..

[6] Wang Y., Lei T., Jiang B., Guo L. (2004). Growth, microstructure and mechanical properties of microarc oxidation coatings on titanium alloy in phosphate-containing solution. Appl. Surf. Sci..

[7] Ao N., Liu D., Zhang X., Liu C. (2019). Enhanced fatigue performance of modified plasma electrolytic oxidation coated Ti-6Al-4V alloy: Effect of residual stress and gradient nanostructure. Appl. Surf. Sci..

[8] Campanelli L., Duarte L.T., da Silva P.S.C.P., Bolfarini C. (2014). Fatigue behavior of modified surface of Ti–6Al–7Nb and CP-Ti by micro-arc oxidation. Mater. Des..

[9] Yoo B., Shin K.R., Hwang D.Y., Lee D.H., Shin D.H. (2010). Effect of surface roughness on leakage current and corrosion resistance of oxide layer on AZ91 Mg alloy prepared by plasma electrolytic oxidation. Appl. Surf. Sci..

[10] Zhu L., Petrova R.S., Gashinski J.P., Yang Z. (2017). The effect of surface roughness on PEO-treated Ti-6Al-4V alloy and corrosion resistance. Surf. Coatings Technol..

[11] Hanjun H., Zhen C., Xingguang L., Xingguo F., Yugang Z., Kaifeng Z., Hui Z. (2018). Effects of substrate roughness on the vacuum tribological properties of duplex PEO/bonded-MoS2 coatings on Ti6Al4V. Surf. Coat. Technol..

[12] Wang J., Huang S., Huang H., He M., Wangyang P., Gu L. (2018). Effect of micro-groove on microstructure and performance of MAO ceramic coating fabricated on the surface of aluminum alloy. J. Alloy Compd..

[13] Nashrah N., Kamil M., Yoon D., Kim Y., Ko Y. (2019). Formation mechanism of oxide layer on AZ31 Mg alloy subjected to micro-arc oxidation considering surface roughness. Appl. Surf. Sci..

[14] Dai W.B., Yuan L.X., Li C.Y., He D., Jia D.W., Zhang Y.M. (2018). The effect of surface roughness of the substrate on fatigue life of coated aluminum alloy by micro-arc oxidation. J. Alloy Compd..

[15] Wang Y., Zhang P., Guo L., Ouyang J.-H., Zhou Y., Jia D. (2009). Effect of microarc oxidation coating on fatigue performance of Ti–Al–Zr alloy. Appl. Surf. Sci..

[16] Gelfi M., Bontempi E., Roberti R., Depero L. (2004). X-ray diffraction Debye Ring Analysis for STress measurement (DRAST): A new method to evaluate residual stresses. Acta Mater..

[17] Härting M., Fritsch G. (1996). Determination of the residual stress state in a natural titanium oxide layer. Acta Mater..

[18] Li C., Dai W., Duan F., Zhang Y., He D. (2017). Fatigue Life Estimation of Medium-Carbon Steel with Different Surface Roughness. Appl. Sci..

[19] Xue W., Deng Z., Chen R., Zhang T. (2000). Growth regularity of ceramic coatings formed by microarc oxidation on Al–Cu–Mg alloy. Thin Solid Films.

[20] Srinivasan P.B., Liang J., Blawert C., Störmer M., Dietzel W. (2009). Effect of current density on the microstructure and corrosion behaviour of plasma electrolytic oxidation treated AM50 magnesium alloy. Appl. Surf. Sci..

[21] Li Z., Yuan Y., Jing X. (2012). Effect of current density on the structure, composition and corrosion resistance of plasma electrolytic oxidation coatings on Mg–Li alloy. J. Alloy Compd..

[22] Zhang Y., Wu Y., Chen D., Wang R., Li D., Guo C., Jiang G., Shen D., Yu S., Nash P. (2017). Micro-structures and growth mechanisms of plasma electrolytic oxidation coatings on aluminium at different current densities. Surf. Coat. Technol..

[23] Tang S., Li Y., Wang Y., Gao Y., Zheng Q., Yi D. (2018). Theoretical study of mechanical and thermodynamic properties of titanium oxides Ti x O y. Mater. Chem. Phys..

[24] Apachitei I., Lonyuk B., Fratila-Apachitei L., Zhou J., Duszczyk J. (2009). Fatigue response of porous coated titanium biomedical alloys. Scr. Mater..

[25] Bai Y., Xi Y., Gao K., Yang H., Pang X., Yang X., Volinsky A.A. (2019). Brittle coating effects on fatigue cracks behavior in Ti alloys. Int. J. Fatigue.

[26] Dai W., Liu Z., Li C., He D., Jia D., Zhang Y., Tan Z. (2019). Fatigue life of micro-arc oxidation coated AA2024-T3 and AA7075-T6 alloys. Int. J. Fatigue.

[27] Leoni A., Apachitei I., Riemslag A., Fratila-Apachitei L., Duszczyk J. (2011). In vitro fatigue behavior of surface oxidized Ti35Zr10Nb biomedical alloy. Mater. Sci. Eng. C.

[28] Khan S.A., Miyashita Y., Mutoh Y., Koike T. (2008). Effect of anodized layer thickness on fatigue behavior of magnesium alloy. Mater. Sci. Eng. A.

